# Persistence and Dissemination Capacities of a *bla*_NDM-5_-Harboring IncX-3 Plasmid in *Escherichia coli* Isolated from an Urban River in Montpellier, France

**DOI:** 10.3390/antibiotics11020196

**Published:** 2022-02-02

**Authors:** Florence Hammer-Dedet, Fabien Aujoulat, Estelle Jumas-Bilak, Patricia Licznar-Fajardo

**Affiliations:** 1 HSM, University Montpellier, CNRS, IRD, 34090 Montpellier, France; florencehammer@hotmail.fr (F.H.-D.); fabien.aujoulat@umontpellier.fr (F.A.); 2 HSM, University of Montpellier, CNRS, IRD, CHU Montpellier, 34090 Montpellier, France; estelle.bilak@umontpellier.fr

**Keywords:** Carbapenemase producing Enterobacterales, plasmid, IncX-3, NDM, carbapenem resistance, one health, water, environment

## Abstract

To investigate the capacities of persistence and dissemination of *bla*_NDM-5_ within *Escherichia coli* and in aquatic environment, we characterized *E. coli* (sequence type 636) strains B26 and B28 isolated one month apart from the same urban river in Montpellier, France. The two isolates carried a pTsB26 plasmid, which sized 45,495 Kb, harbored *bla*_NDM-5_ gene and belonged to IncX-3 incompatibility group. pTsB26 was conjugative in vitro at high frequency, it was highly stable after 400 generations and it exerted no fitness cost on its host. *bla*_NDM-5_harboring plasmids are widely dispersed in *E. coli* all around the world, with no lineage specialization. The genomic comparison between B26 and B28 stated that the two isolates probably originated from the same clone, suggesting the persistence of pTsB26 in an *E. coli* host in aquatic environment.

## 1. Introduction

Antimicrobial resistance (AMR) occurs worldwide and the World Health Organization has identified it as one of the three main threats to human health [1,2]. Due to the overuse and misuse of antimicrobials, selective pressure exerted on bacteria significantly enhances AMR, with consequences for antimicrobial treatments failures [3]. At first neglected, the environment is now considered as a main player in the emergence and diffusion of AMR. Water plays a major role in interconnecting different ecosystems such as humans, animals, soils and hydrosystems [4,5,6,7,8]. Urban and rural surface waters constitute hotspots for exchanges among microorganisms of human and environmental origin, which are all subject to strong selection pressures due to diverse pollutions [3,5]. β-lactams are by far the most widely consumed antibiotics worldwide [9], and among β-lactams, carbapenems are last resort treatment for multidrug-resistant bacterial infections [10,11,12]. Several studies reported the occurrence of Carbapenemase Encoding Genes (CEGs) in aquatic environments [6,7,8]. However, there is a lack of studies on the persistence and dissemination capacities of CEGs and bacteria carrying CEGs in waters.

In 2009, a New Delhi Metallo-β-lactamase-1 (NDM-1) encoding gene was first identified in a carbapenem-resistant *Klebsiella pneumoniae* involved in a urinary tract infection [13]. Since then, NDM-producing *Enterobacterales* have spread rapidly in humans and animals, and in various environments [14,15,16,17]. NDM enzymes belong to class B β-lactamases, which displays a broad lysis spectrum, hydrolyzing almost all β-lactams [7]. Until now, 29 variants of NDM have been described [18]. Several plasmids harboring *bla*_NDM_ genes have been identified. Among them, plasmids of the incompatibility group IncX-3 are frequently associated with *bla*_NDM-5_ [14,19,20,21,22,23,24]. The IncX-3 plasmids group gathers self-transmissible plasmids with a narrow host spectrum restricted to *Enterobacterales* [25]. Characterizing these plasmids would be contributive for understanding their role in environmental AMR issue.

Several clinical, animal and environmental strains containing IncX-3 plasmids carrying *bla*_NDM-5_ have been isolated in China, South East Asian countries [26,27,28] and French urban waters [29]. Here, we described pTsB26, an IncX-3 plasmid encoding *bla*_NDM-5_ gene carried by two strains of *E. coli* isolated from an urban river one month apart. We studied the capacities of dissemination and persistence of pTsB26 by in vitro assays and the fitness cost associated with pTsB26 carriage. Metadata analysis was performed in order to identify common themes in *bla*_NDM-5-_harboring IncX-3 plasmids and to gain insights in IncX-3 success in *E. coli* population. The worldwide circulation of *bla*_NDM-5-_harboring IncX-3 plasmids in *E. coli* and in various environments as well as the high stability in host bacteria observed herein could explain the contribution of this type of plasmid in the global dissemination of carbapenemase genes.

## 2. Results

### 2.1. Characteristics of the Two NDM-5 Producing E. coli Isolates B26 and B28

B26 and B28 *E. coli* were isolated in the urban river of Font d’Aurelle, in August and September 2015, respectively. They both displayed high level of carbapenem resistance, with minimum inhibitory concentrations (MIC) over 15 mg/L for ertapenem, meropenem and imipenem.

The B26 genome is 4927 Mb and includes four plasmids belonging to IncFIA/FIB, IncFII, IncQ1 and IncX-3 incompatibility groups. The inventory of antimicrobial resistance genes identified *bla*_NDM-5_, *bla*_TEM-1B_ and *bla*_SHV-12_ (previously identified as *bla_SHV-5_* by multiplex PCR GeneXpert Cepheid). Genome of B28 is 4858 Mb, including a unique IncX-3 plasmid and *bla*_NDM-5,_ was the only antimicrobial resistance gene detected.

Both isolates were affiliated to the sequence type (ST) 636 (B2 phylogroup) by in silico multilocus sequence typing. Whole-genome alignment and comparison showed no large indels. Only 21 variations between B26 and B28 genomes were identified, including 19 single nucleotide polymorphisms (SNPs) and two multi-nucleotide polymorphisms (four nucleotides). Among the 21 variations, six variations were related to hypothetic encoding sequences, four of them corresponding to synonymous mutations and two to missense mutations.

### 2.2. Characteristics of the bla_NDM-5_-Harboring IncX-3 Plasmids

Transformation assays in *E. coli* TOP10 with plasmids extracted from B26 and B28 were successful and gave two transformants, TsB26 and TsB28. These two transformants displayed high level of resistance to carbapenems (MIC ≥ 7.5 mg/L whatever the carbapenem). They were positive to specific PCRs anchored in *pir* gene (i.e., specific of IncX-3 plasmid) and *bla*_NDM_. PCR tests indicated that both transconjugants did not contain any other plasmid.

**Figure 1:**
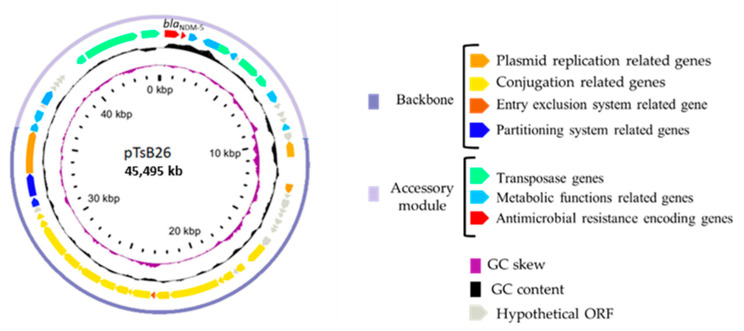
pTsB26 plasmid representation.

Genomic sequences of TsB26 and TsB28 were aligned and compared with those of *E. coli* TOP10. TsB26 presented a unique additional sequence onto a single contig corresponding to IncX-3 plasmid. The extremities of this contig were *bla*_NDM-5_ and IS*Aba125*, corresponding to a plasmid region already sequenced [29]. Complete identity was observed between pTsB28 and pTsB26 encoding *bla*_NDM-5_ plasmids. So, the plasmid was called pTsB26 from then on. pTsB26 sizes 45,495 kb with a GC content of 46.5%. It belongs to IncX-3 plasmid incompatibility group. Nucleotide sequence analysis revealed 57 predicted open reading frames corresponding to 57 encoding genes (Figure 1). Alignment of pTsB26 with IncX3-plasmid conserved backbone, described by Liakopoulos [30], showed that pTsB26 backbone was typical of IncX-3 group. It is approximately 25 kb and includes encoding genes for replication (*pir* and *bis*), entry exclusion (*eex*), plasmid stability (*par*AB, *topB* and *hns*) and conjugative transfer (*pilX1–11* and *taxA-C*) [30]. The accessory module of about 20 kb contains *bla*_NDM-5,_ which is preceded by IS*3000* and IS*Aba125* and followed by *ble*_MBL_ (bleomycin resistance gene), *trp*F (N-5′phosphoribosylanthranilate isomerase)*, dsb*D (disulfide oxidoreductase) and *umu*D (encoding a protein implicated in the SOS system).

BLASTn analysis showed that pTsB26 displayed nearly the same sequence (45494/45495 bp) as the plasmid pEC7-NDM-5 (accession number: MH347484) found in a *E. coli* strain isolated from dog in South Korea. A high homology (45475/45495 bp) was also observed with the well characterized IncX-3-blaNDM5 pEC463-NDM5 plasmid (accession number: MG545911) in an *E. coli* clinical strain from China [31].

### 2.3. Conjugative Transfer Success of pTsB26 In Vitro

Conjugative transfer rate of pTsB26 was studied by mating assays, with B26, B28, TsB26 and TsB28 as donor strains and XL1-Blue *E. coli* as receptor. All strains successfully transferred pTsB26, with high transfer rates (Table 1), and all transconjugant strains displayed carbapenem resistance (MIC ≥ 7.5 mg/L).

### 2.4. In Silico Population Study of bla_NDM-5_-Harboring IncX-3 Plasmids among E. coli Species

A genomes dataset was constructed with 28 complete genomes of *E. coli-*carrying *bla*_NDM-5_ on an IncX-3 plasmid and the genomes of B26 and B28 strains. In silico sequence types, phylotypes and metadata associated with the genomes are presented in Table 2. More than 96% of the genomes belong to A, B1 and C phylogroups. B26 and B28 were the only genomes of the B2 phylogroup in the dataset. The 31 genomes corresponded to strains isolated from diverse origins: humans (*n* = 20), environment (*n* = 6) and animals (*n* = 4).

**Figure 2:**
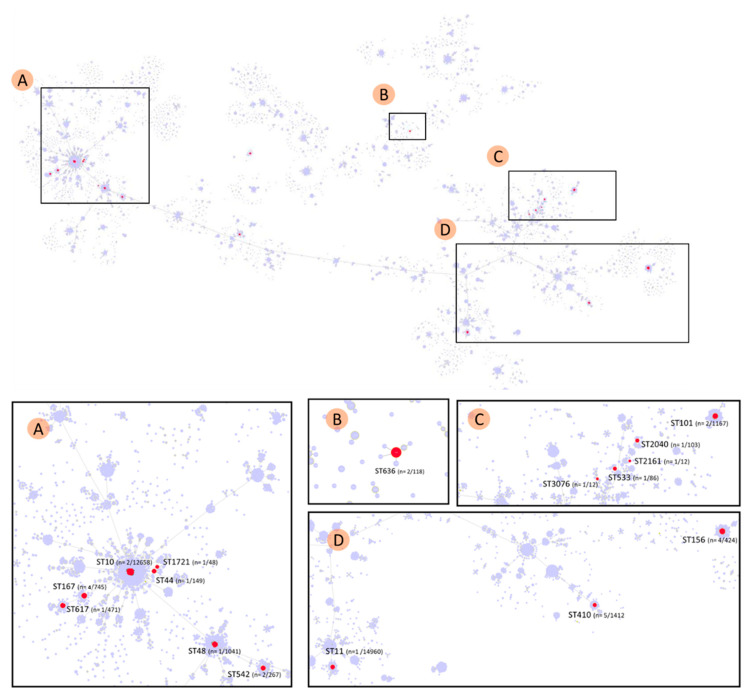
goeBURST diagram of *E. coli* genomes carrying a *bla*_NDM-5_ encoding IncX-3 plasmid within a global population of *E. coli* established on 178 776 strains available in the EnteroBase database. Each node corresponds to a Sequence Type (ST). The size of the node is scaled to the number of genomes of that ST. Nodes linked between them present one allele in common among the 7 genes considered in the MultiLocus Sequence Type scheme. Red nodes correspond to STs for which genomes with a *bla*_NDM-5_ encoding IncX-3 plasmid was identified; the proportion in the ST of genomes containing the plasmid is noted in parentheses.

In order to study the distribution of *E. coli* encoding *bla*_NDM-5_ on IncX-3 plasmids within the whole *E. coli* populations, we reconstructed genetic links by goeBURST analysis. The dataset of 30 genomes was matched with 178 776 available genomes of *E. coli* (11 058 STs) in the dataset EnteroBase (13 July 2021) (Figure 2). The genomes of *E. coli-*carrying *bla*_NDM-5_ IncX-3 plasmid spread out in 16 STs with no obvious lineage specialization. However, about half of the genomes (41.9%) belong to the CC10. This CC is a major sub-population in *E. coli* because it gathers 11.85% of the STs available in EnteroBase. The other genomes are scattered in the overall *E. coli* population structure. B26 and B28 belonged to ST636, which forms the CC636 together with 4 related STs. CC636 was relatively isolated in the *E. coli* population and gathers 451 strains (0.25%) of the 178,776 strains of EnteroBase.

### 2.5. Stability and Fitness Cost of pTsB26 on B26 and B28 E. coli

**Figure 3:**
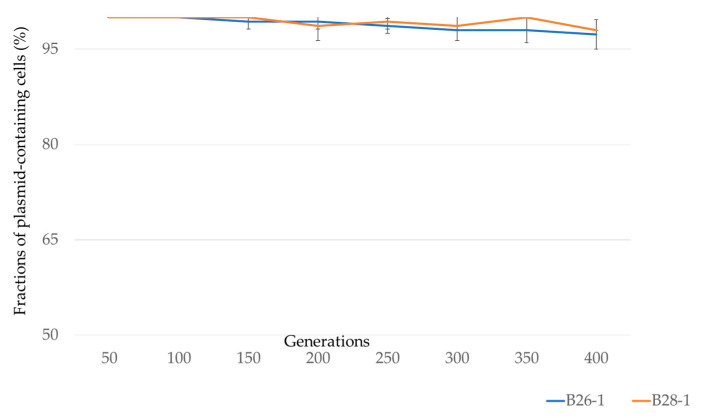
Stability of pTsB26 in B26 and B28.

To evaluate the stability of the pTsB26 plasmid in B26 and B28, strains were passaged daily for 40 days without antibiotic selection. pTsB26 is highly stable, with more than 96% of plasmid containing cells after approximately 400 generations (Figure 3).

These assays allowed one to isolate strains without pTsB26: B26ΔpJ40 and B28ΔpJ19, respectively, isolated at day 40 and day 19 of the experiment. In parallel, two strains containing pTsB26 plasmid (B26J40a and B28J19a) were isolated the same day as B26ΔpJ40 and B28ΔpJ19. These strains, used as control, were thereafter called “LB-adapted” strains, with the letter “a” at the end of the strain name.

Growth kinetics assays were performed for the strains with (ancestral strains and LB-adapted strains) and without pTsB26 plasmid (Table 3). Growth rates of B26, B26ΔpJ40 and B26J40a did not vary significantly (*p* > 0.05), suggesting that the carriage of pTsB26 did not produce fitness lost for B26. Of note, B26ΔpJ19 and B26J19a contained the other plasmids IncFIA/FIB, IncFII and IncQ1 as the ancestral B26. On the other hand, growth rates of ancestral B28 or LB-adapted B28 (B28J19a) were equivalent and significantly better than observed for B28 cured for pTsB26 (*p* < 0.05). This last observation suggests that pTsB26 could provide fitness advantage even to its host, without selective pressure.

## 3. Discussion

The spread of NDM-5 variant has been extensively described in hospital environments, including hospital sewage water [32,33] and in wastewater [34]. In France, in 2020, about 20% of carbapenemase-producing enterobacteria isolated from clinical samples were NDM-producers [35]. Among them, more than 35% were NDM-5 variant. Its emergence and successful spread are worth emphasizing as this variant represented only 5% of NDMs reported in 2013, 15% in 2016 and more than 30% in 2017, with stabilization since this date [35].

Focusing on hydric environments, this variant has been found in various environments such as urban waters [29,36]; rivers and lakes [8,37]; sediments and soil [38]; and seawater [8]. Various plasmid incompatibility groups carrying *bla*_NDM-5_ have been reported, with IncX-3 and IncF being among the most prevalent [8,27,34,37,38,39,40].

Here, we report the description of the *bla*_NDM-5_ carrying plasmid pTsB26 from two *E. coli* ST636 isolated from an urban river in Montpellier, France [29]. IncX-3 plasmids have a narrow host spectrum restricted to *Enterobacterales* species [25,28,41]. Their association with antimicrobial resistance has been documented worldwide [28,41,42,43,44,45,46,47,48,49].

The involvement of IncX-3 plasmids in the dissemination of *bla*_NDM_ genes was first described in the 2000′s [43]. It became quickly prevalent within *Enterobacterales* all around the world, with predominance in Asia [27,28,41,42,44,45,46,47,49,50]. Here, we show that *bla*_NDM-5-_harboring IncX-3 plasmids are widely disseminated in the species *E. coli* in all the continents, with no lineage specialization (Figure 3). This strongly suggests horizontal spread within *E. coli* and proves the important implication of IncX-3 plasmid in the success of *bla*_NDM-5_ gene in this species.

Only 21 variations (19 SNPs and 2 multi-nucleotide polymorphisms) were detected between B26 and B28 isolated in the same urban aquatic environment a month apart. The scarce genomic differences strongly suggest their clonal origin. Clonal strains have certainly persisted for at least one month in the river, with iterative input being highly unlikely but not excluded. This hypothesis is strongly supported by the fact that the two isolates carried the same plasmid pTsB26 (100% nucleotide identity). Interestingly, the IncFIA/FIB, IncFII and IncQ1 plasmids carried by B26 (isolated in August 2015) were absent from the genome of B28 (isolated in September 2015). Only pTsB26 has persisted in the *E. coli* strain isolated in September, showing that pTsB26 is stable in B26 and B28 isolates, relative to other plasmids in the same strains. This in situ observation was verified by in vitro evolution experiments, demonstrating the longtime persistence of the plasmid after 400 generations in B26 and B28 isolates (Figure 3). Several factors could explain the stability of pTsB26 in the cell lineage. First, like the other IncX-3 plasmids, pTsB26 harbors the widespread partitioning system ParAB. This system limits the number of segregant cells during cell division, ensuring the correct inheritance of the plasmid to the daughter cells [51]. Moreover, the conjugative traits of pTsB26 allow for infection of segregant cells and thus limit their number. Thus, harboring pTsB26 does not reduce the fitness of B26 and B28 (Table 3) and enhances the growth of B28. It is generally admitted that plasmids cause a fitness burden on their bacterial host [52,53,54,55] and that the plasmid could be quickly eliminated from its host. High stability of IncX-3 plasmids has already been reported in *Enterobacterales* transconjugant strains [28,56]. The absence of fitness cost of IncX-3 plasmids could be explained by their small size [25] and by the presence of transcriptional regulator H-NS like protein [28,30,57,58,59,60]. The observed high stability of pTsB26 has potential due to its high conjugation frequency, to a low rate of segregational loss and due to the fact it does not pass a fitness cost onto the bacterial host.

The isolates B26 and B28 are the unique representants of ST636 in the studied dataset of *E. coli-*carrying *bla*_NDM-5-_encoding IncX-3 plasmid (Table 2). However, in their study, Kumwenda et al. reported two *E. coli* ST636 clinical isolates that carried *bla*_NDM-5_ onto a plasmid not affiliated to an incompatibility group [61]. EnteroBase reported 118 isolates belonging to ST636 (Figure 3). They were isolated in different countries (all continents are represented) and in environmental and clinical samples. Other studies reported the occurrence of ST636-producing ESBLs in clinical and environmental samples [62,63,64], suggesting a generalist trait for this ST.

Persistence of strains or STs carrying CEGs on self-transmissible plasmid such as pTsB26 in aquatic environment is of concern. Aquatic environment contains diverse autochtonous bacteria, including *Enterobacterales* (e.g., *Enterobacter* sp. [65] or *Raoultella* sp. [66]), which can exchange and receive IncX-3 plasmids [27,28,41]. These autochtonous bacteria can constitute an environmental reservoir and shuttles for *bla*_NDM_ genes. Aquatic environments are strongly linked with anthropic activities, and during recreational activities, after flood episodes or by alimentation, humans can become exposed to bacteria from aquatic environments [7,67,68,69,70]. Thus, if water contains carbapenemase-producing-bacteria such as B26 or B28 or other autochtonous bacteria, and has acquired the plasmid by horizontal gene transfer, it represents a risk for human health (i) directly by causing antimicrobial-resistant bacterial infections [71] and (ii) indirectly by participating in the dissemination of *bla*_NDM-5_ on the occasion of gut colonization [71,72], or transit. These resistant bacteria can transfer the plasmid to host microbiota bacteria, making a “shuttle” between aquatic environment and humans [7,71].

We described for the first time the in situ persistence of a *bla*_NDM-5_harboring IncX-3 self-conjugative plasmid in an *E. coli* lineage in an aquatic environment. This study underlines, once again, the importance of investigations into environmentally emerging, resistant bacteria. Beside genomics, testing genetic transfer and resistance stability by in vitro evolution is a proxy for diffusion and persistence in natural environment. In addition to the strategy proposed in this study, experiments of resistance genes transfer to waterborne autochthonous bacteria in microcosm would be interesting to conduct for a better description of the resistance reservoirs and the conditions influencing these reservoirs. On another hand, rapid alerts on environmentally emerging antimicrobial resistance are needed for rapid responses to resistance with public health concern. For this, efficient surveillance of AMR in environment should be undertaken. This is one of the challenges of the current national and international projects aiming to limit global AMR outbreak.

## 4. Materials and Methods

### 4.1. Escherichia coli Strains

*E. coli* strains B26 and B28 were isolated from water sampled at the same site of the urban river Font d’Aurelle in the city of Montpellier (N43.62711 E003.85316), France. They were isolated in August and September 2015, respectively [29].

Transformant strains, TsB26 and TsB28, were obtained by transformation experiments (see Section 4.2.).

Strains B26ΔpJ40, B28ΔpJ19, B26J40a and B28J19a were obtained during plasmid stability assays (see Section 4.3.).

### 4.2. Transformation and Conjugation Assays

Plasmid DNA extraction was done using the NucleoSpin Plasmid Kit (Macherey-Nagel, Allentown, PA, USA). Plasmid extracts were used for transformation assays using One Shot TOP10 chemically competent *E. coli* (Invitrogen, ThermoFisher Scientific, Paisley, UK) as recipient cell.

Conjugation experiments were performed using non-competent XL1-Blue *E. coli* MRF’, a recipient strain resistant to tetracycline and sensitive to meropenem. Briefly, donor (B26, B28 and transformants TsB26 and TsB28) and recipient strains were grown overnight at 37 °C in Luria Bertani (LB) broth supplemented (donor strains) or not (recipient strain) with ertapenem (4 mg/L). Cells were washed from antibiotic and resuspended in LB broth, and each donor strain suspension was mixed (1:1 ratio) with the receptor strain. 200 µL of each mix was deposited onto nitrocellulose membrane, itself stuck on LB agar media and incubated at 37 °C during 24 h. Transconjugants were selected by plating the bacteria from the nitrocellulose membrane onto LB agar plates supplemented with ertapenem (4 mg/L) and tetracycline (12 mg/L). The conjugative frequencies were determined by calculating the transfer rate (ratio transconjugant/donor).

The presence of *bla*_NDM_ and *pir* (encoding the IncX-3 plasmid-specific Pir protein) in selected transconjugants and transformants was assessed by specific PCRs [14,73].

### 4.3. Evaluation of Plasmid Stability

Strains B26 and B28 were grown overnight at 37 °C in 10 mL of LB broth supplemented with ertapenem (4 mg/L). Bacterial cells were washed from antibiotic by centrifugation, the pellet was resuspended in 1 mL of LB broth and 10 mL of fresh LB broth without antibiotic was spiked with 10 µL of the bacterial suspension and incubated 24 h at 37 °C in a shaking water bath. Serial passages of 10 µL of overnight culture to 10 mL of fresh LB broth were done daily. One passage corresponded approximatively to 10 generations of growth. Every 50 generations, samples were diluted and plated on LB agar plates. Then, 50 colonies from each lineage were screened on LB agar plates supplemented or not with ertapenem (4mg/L) to determine the fraction of plasmid-containing cells. The lack of plasmid was confirmed by the absence of *bla*_NDM_ and *pir* genes with specific PCRs, and these strains (B26ΔpJ40 and B28ΔpJ19) were harvested for fitness cost assays. Parallelly, strains from the same generation, carrying pTsB26 plasmid, were harvested as controls (B26J40a and B28J19a). Experiments were done in triplicate.

### 4.4. Fitness Cost of Plasmid Carriage

Growth of strains carrying (B26, B28, B26J40a and B28J19a) or not (B26ΔpJ40 and B28ΔpJ19) pTsB26 plasmid were measured at 37 °C in LB broth without antibiotics using a CLARIOstar Plus microplate reader (BMG, Labtech). Every 15 min, the microplate was shacked at 200 rpm during 20 s, and optical density was measured at 600 nm. Growth rates were calculated according to Sandegren et al. [74].

### 4.5. Carbapenem Susceptibility Testing

Susceptibility to carbapenems of B26, B28, TsB26, TsB28, B26J40a, B28J19a, B26ΔpJ40 and B281ΔpJ19 was assessed by determining the Minimal Inhibitory Concentration (MIC) in liquid media [75] for ertapenem, meropenem and imipenem. *E. coli* strain ATCC 25922 was used as control strain, as recommended by the CA-SFM (https://www.sfm-microbiologie.org/2021/04/23/casfm-avril-2021-v1-0/, accessed on 28 September 2021).

### 4.6. In Silico Analysis

#### 4.6.1. DNA Extraction and Whole-Genome Sequencing

Genomic DNA of B26, B28, TsB26, TsB28 and *E. coli* TOP10 was extracted using the MasterPure™ purification kit (Lucigen, Middleton, WI, USA). High-throughput genome sequencing was carried out at the Plateforme de Microbiologie Mutualisée (P2M, Institut Pasteur, Paris, France). DNAs were processed for sequencing with Illumina systems (libraries using the Nextera XT DNA Library Prep kit and sequencing with the NextSeq 500 system). Paired-end reads were submitted to pre-processing using fqCleaner and to de novo assembly using SPAdes v3.12.0 [76] with k-mer lengths 21, 33, 55 and 77. The raw data and assemblies have been deposited in GenBank under the BioProject accession number PRJNA796954.

#### 4.6.2. Genotyping Methods

B26 and B28 strains were genotyped by Clermont typing [77] using the in silico tool EzClermont [78] and by MultiLocus Sequence Typing (MLST) using the Achtman scheme (https://pubmlst.org/data, accessed on 28 September 2021).

#### 4.6.3. Plasmid Sequence and Annotation

The sequence of the plasmid pTsB26 was deduced from the alignment of the genomes of TsB26 and TsB28 with that of *E. coli* TOP10 using the ProgressiveMauve algorithm of Mauve software [79]. Identification of plasmid incompatibility group was done with PlasmidFinder [80]. Plasmid annotation was performed with Prokka v1.14.6 [81] with default parameters through the Galaxy platform (v4.6.0 + galaxy0) [82], and the plasmid sequence was manually curated; plasmid-specific genes were named according to Thomas et al. recommendations [83].

For ORF annotation as hypothetical protein, functional prediction was performed with default settings using NCBI BLASTp [84], InterProScan [85] and Pfam [86] servers. Identification of antimicrobial resistance genes was done using ResFinder 4.1 [87]. pTsB26 representation was done using genomeVx [88].

#### 4.6.4. Comparative Genomics of B26 and B28 Genomes

Genomes of B26 and B28 were first aligned with the ProgressiveMauve algorithm of Mauve software. Snippy v4.6.0 (https://github.com/tseemann/snippy, accessed on 20 November 2021) was used to detect both substitutions and insertions/deletions (indels) between B26 and B28 genomes. Snippy was run on the Galaxy platform (v4.6.0 + galaxy0) [82] with the default parameters.

#### 4.6.5. Distribution of IncX3-*bla*_NDM_ Plasmids in *E. coli* Population

In order to select *E. coli* genomes carrying *bla*_NDM-5_ on an IncX3 plasmid, complete genomes available on NCBI database (6 April 2021) were investigated using BLASTn tool [84]. *bla*_NDM-5_ and *pir* gene of pTsB26 sequences were used, and genomes carrying the 2 genes (minimum homology 100% and 92%, respectively) on the same replicon were selected. Metadata associated with each genome were collected, and ST were determined in silico using the MLST 2.0 [89]. STs (Achtman MLST scheme) associated with selected genomes and B26 and B28 strains were compared to general *E. coli* population using EnteroBase database [90] (178,776 strains the 13 July 2021) and the goesBURST algorithm (single locus variant level) of PHYLOViZ 2.0 software [91]. Plasmid homologies were determined by BLASTn analysis [84] against the NCBI nr/nt database with default parameters.

### 4.7. Statistical Assays

Statistical analysis of growth rates was performed using Student’s t-test. All statistics were made using the GraphPad Prism software V 5.03. Test results were considered as statistically significant when the associated *p*-value was less than 0.05.

## Figures and Tables

**Table 1:** Conjugative transfer rates of pTsB26 from different donor strains to *Escherichia coli* XL1-Blue receptor.

Donor Strains	Conjugative Frequency
B26	2.09 × 10^−3^
B28	4.81 × 10^−4^
TsB26	3.52 × 10^−3^
TsB28	3.76 × 10^−3^

**Table 2:** Characteristics of *E. coli* genomes carrying a *bla*_NDM-5_ IncX-3 plasmid.

Strain	Accession Number	ST	Phylotype	Country	Source Type	Source Niche	Year
CRE1493	CP019071	167	A	China	rectal swab	*homo sapiens*	2013
165	CP020509	101	B1	USA	abdominal	*homo sapiens*	2015
CREC-591	CP024821	101	B1	South Korea	peritoneal fluid	*homo sapiens*	2015
WCHEC025943	CP027205	410	C	China	wastewater	environment	2017
WCHEC005784	CP028578	617	A	China	rectal swab	*homo sapiens*	2014
135	CP028632	11	E	Canada	NA	livestock	2006
ECCRA-119	CP029242	156	B1	China	stools	dog	2017
CH613	MCRE01000001	10	A	China	urine	*homo sapiens*	2015
GSH8M-2	NZ_AP019675	542	A	Japan	wastewater treatment plant	environment	2018
WP8-S18-CRE-02	NZ_AP022245	542	A	Japan	wastewater treatment plant	environment	2018
TUM18781	NZ_AP023205	2040	B1	Japan	NA	*homo sapiens*	2018
YJ3	NZ_AP023226	10	A	Myanma	stools	*homo sapiens*	2018
WCHEC005237	NZ_CP026580	167	A	China	rectal swab	*homo sapiens*	2014
SCEC020001	NZ_CP032426	410	C	China	urinary tract	*homo sapiens*	2016
SCEC020022	NZ_CP032892	156	B1	China	stools	*homo sapiens*	2016
WCHEC020031	NZ_CP033401	410	C	China	NA	*homo sapiens*	2016
L37	NZ_CP034589	48	A	China	rectal swab	*homo sapiens*	2018
L65	NZ_CP034738	3076	B1	China	NA	*homo sapiens*	2018
SCEC020026	NZ_CP034958	410	C	China	NA	*homo sapiens*	2016
WCHEC020032	NZ_CP034966	410	C	China	NA	*homo sapiens*	2016
WCHEC025970	NZ_CP036177	167	A	China	NA	*homo sapiens*	2017
L725	NZ_CP036202	2161	B1	China	stools	*homo sapiens*	2018
EC-129	NZ_CP038453	167	A	Japan	sputum	*homo sapiens*	2018
GZ04-0086	NZ_CP042336	44	A	China	stools	*homo sapiens*	2018
GZEC065	NZ_CP048025	156	B1	China	blood	*homo sapiens*	2017
pV11-19-E11-025-038	NZ_CP049050	1721	A	South Korea	NA	dog	2019
3R	NZ_CP049348	156	B1	China	NA	poultry	2015
SFE8	NZ_CP051219	533	B1	China	stools	pork	2019
B26	B26	636	B2	France	urban water	environment	2015
B28	B28	636	B2	France	urban water	environment	2015

ST, Sequence Type; NA, Not Available.

**Table 3:** Growth rates of strains with and without the pTsB26 plasmid.

Strain	µmax (h^−1^) (±sd)
B26	0.43351667 (±0.02560949) ^a^
B26ΔpJ40	0.4441375 (±0.02775884) ^a^
B26J40a	0.44380714 (±0.03785335) ^a^
B28	0.43683889 (±0.0217273) ^a, b^
B28ΔpJ19	0.40048824 (±0.0333677) ^c^
B28J19a	0.43095 (±0.03460016) ^a, b^

sd, standard deviation; values with a different letter (a,b,c) are significantly different at *p* < 0.05 (Student’s t-test).
